# Chances of Liver Transplantation in a Patient With Transaldolase Deficiency Complicated by Hepatopulmonary Syndrome

**DOI:** 10.7759/cureus.35150

**Published:** 2023-02-18

**Authors:** Ebtehal Fallata, Aisha M Alamri, Hadeel A Alrabee, Abdulhadi A Alghamdi, Ameera Alsaearei

**Affiliations:** 1 Department of Pediatrics, East Jeddah General Hospital, Jeddah, SAU; 2 Department of Pediatrics, Division of Gastroenterology, East Jeddah General Hospital, Jeddah, SAU; 3 Department of Pediatrics, Division of Cardiology, East Jeddah General Hospital, Jeddah, SAU

**Keywords:** liver transplantation, taldo1, deficiency, transaldolase, cirrhosis, syndrome, hepatopulmonary, hepatosplenomegaly

## Abstract

Eyaid’s syndrome or Transaldolase Deficiency (TD) (OMIM 606003) is a rare autosomal recessive inborn error of metabolism. In this report, we describe the case of an eight-year-old Saudi girl with a history of hepatosplenomegaly since infancy, who presented to the emergency department for a short history of cough and worsening cyanosis. She had growth retardation, facial dysmorphia, cardiac defect, neutropenia, and thrombocytopenia, besides hepatosplenomegaly. A thorough investigation led to the diagnosis of hepatopulmonary syndrome and whole exome sequencing showed a homozygous frameshift variant in the TALDO1gene, c.793del, p.Gln265fs. Thus, the patient was diagnosed with TD complicated with hepatopulmonary syndrome, and the indication of liver transplantation was discussed.

## Introduction

Eyaid’s syndrome or Transaldolase Deficiency (TD) is a rare autosomal recessive inborn error of metabolism, which is registered under phenotype MIM OMIM# 606003. The genetic defect lies in the pentose phosphate pathway and is caused by a mutation in the transaldolase 1 gene on chromosome 11p15 [1]. The condition was named after Doctor Wafa Al Eyaid, a Saudi doctor, subsequent to her research work on 12 children from six families, who presented with various cardiac defects and skin and facial dysmorphic disorders, in association with hepatosplenomegaly, anemia, and thrombocytopenia [2]. However, the initial case of TD was described in 2001, by Verhoeven et al., and concerned a 10-year-old girl of consanguineous Turkish parents, who presented with liver cirrhosis of unknown origin in early childhood [3]. Since then, few other cases of TD have been reported in the literature [4].

TD disease presentation varies between patients, combining multiple scenarios including end-stage liver disease, renal tubular dysfunction, coagulopathy, anemia, thrombocytopenia, congenital heart abnormalities, and hormonal disorders [5]. On the other hand, TD is distinct in its dysmorphic features that include a triangular-shaped face, low-set ears, wide mouth, and thin lips, with the most prominent skin feature being wrinkled skin (cutis-laxa) [6]. The diagnosis can be made by the detection of polyols and sugars in urinary excretion and serum [7]; however, whole exome sequence analysis is confirmatory [8].

Hepatopulmonary syndrome (HPS) is a complication of liver disease, that is formed by the triad: hepatopathy, hypoxemia, and intrapulmonary vasodilation [9]. On a controversial note, there is heterogeneity in the reported types of liver disease and diagnostic tools for HPS. HPS leads to chronic oxygen supplementation dependency altering the child’s quality of life and can evolve into respiratory failure and death if left untreated. Liver transplantation is the only effective curative treatment of HPS known to date [9]. Only three cases of HPS diagnosed in the context of TD have been described. Herein, we report a case of an eight-year-old female patient with TD that was associated with HPS.

## Case presentation

An eight-year-old Saudi girl presented to the emergency department with a one-week history of cough and worsening cyanosis. She was known to have hepatosplenomegaly since infancy, with no definitive diagnosis. At that time, she was referred to a tertiary hospital for further investigations, but the family discharged her against medical advice, leading to a loss of follow-up for eight years.

The patient was born at term through uneventful, spontaneous vaginal delivery, with a 2000-g birth weight. The mother reported a 14-day admission to the neonatal intensive care unit for intrauterine growth restriction, during which splenomegaly and thrombocytopenia were observed. An echocardiography was then performed, showing a congenital heart disease, with no further details available to the mother on the day of the presentation. The subsequent years of life were marked by multiple admissions for anemia and thrombocytopenia, which were managed with blood and platelet transfusions without a particular etiology reported. No surgeries were performed. The family history showed non-consanguineous parents with five other healthy siblings, one is having minimal dysmorphism but was clinically stable. However, two of the patient’s cousins were reported to have undergone liver transplantation due to undefined reasons.

On examination, the patient appeared underweight with dysmorphic traits including a triangular face, frontal bossing, small chin, thin lips, and a wide mouth. She was, however, conscious, oriented, and not in distress. She had central cyanosis, grade 4 digital clubbing, and dry skin, with multiple spider naevi over the cheeks and dilated neck veins. Vital signs were as follows: heart rate 92/min; respiratory rate 25/min; blood pressure 104/69 mmHg; temperature 36.5 °C; peripheral oxygen saturation 68% sitting and 80% in the supine position, which increased to 87% with a 10-liter oxygen mask. She weighed 19 kg (far below the third centile) and stood at a height of 130 cm (at the fifth centile). Pulmonary examination showed good breath sounds bilaterally, while cardiovascular auscultation revealed a systolic murmur mainly heard at the aortic area with normal first and second heart sounds. The abdomen was mildly distended, with a non-tender, firm, round-edged hepatomegaly with a span of 18 cm, along with a massive splenomegaly palpated at 8 cm below the costal margin. There were no ascites on physical examination. Examination of the central nervous system found an active normal gait with a Glasgow coma scale of 15/15.

The results of laboratory studies which included complete blood count (CBC), coagulation tests, liver function tests, and lipid levels are summarized in Table 1. Venous blood gas analysis showed hypocapnic alkalosis (blood pH: 7.46, PCO_2_: 27.8 mmHg, PO_2_: 77 mmHg, HCO_3_-: 21.8 mEq/L). Serum vitamin D 25 (OH) level was (18 ng/dL; normal range: 20-50 ng/mL). The remaining tests were within reference ranges; these included electrolytes, renal function tests, thyroid testing, blood glucose (97 mg/dL), ammonia (66 ug/dL; reference range 31-123 ug/dL), lactate (0.9 mmol/L; reference range 0.5-2.2 mmol/L), alpha-fetoprotein (6.3 ng/mL; reference range: 1.09-8.04 ng/mL), and a normal glucocerebrosidase enzyme activity.

**Table 1:** Laboratory findings INR: international normalised ratio; ALT: alanine transaminase; AST: aspartate aminotransferase; GGT: gamma-glutamyl transferase; TG: triglyceride

Test	Result	Reference range
White blood cells	4 10^^^3/uL	6-16
Absolute neutrophil count	1.3 10^^^3/uL	2-8
Hemoglobin	14.7 g/dL	11.5-15.5
Platelet	77 10^^^3/uL	150-450
Prothrombin time	15.8	11.5-15
INR	1.18	0.9-1.1
Partial thromboplastin time	33.9	25-40
ALT	18 U/L	5-55
AST	55 U/L	5-34
Albumin	3.8 g/dL	3.8-5.4
GGT	42 U/L	9-36
Alkaline phosphatase	205 U/L	156-369
Total bilirubin	2.38mg/dL	0.2-1.2
Direct bilirubin	0.77 mg/dL	
Lactate dehydrogenase	271 U/L	125-243
TG	92 mg/dL	<150
Cholesterol	104 mg/dL	<170

In viewing radiological investigations, a chest x-ray showed bilateral pulmonary hyperinflation with no cardiomegaly. Abdominal ultrasound revealed an enlarged liver (markedly prominent caudate lobe) with no perceived focal lesions or gallbladder/biliary tract anomalies. The spleen was also hypertrophied measuring about 13 cm in length. The kidneys were marked as normal. Due to the patient’s uncooperativeness, the liver vasculature could not be assessed on ultrasound. Computed tomography of the abdomen and pelvis demonstrated severe hepatomegaly with irregular liver contours consistent with parenchymal liver disease, and the caudate lobe was disproportionately large. In addition to these findings, a small hypodense focal lesion measuring about 7 mm for the longest diameter and localized in segment number 7 was found. Moreover, signs of portal hypertension were identified including severe congestive splenomegaly, portosystemic shunt to the left renal vein, and minimal free fluid in the pelvis indicating intraperitoneal fluid. Lungs were radiologically unremarkable on chest computed tomography.

An echocardiogram was also performed without any remarkable anomalies and was completed by an agitated saline transthoracic echocardiography to rule out pulmonary arteriovenous malformation. The agitated saline testing was carried out twice; both times positively showed the existence of an arteriovenous malformation.

Altogether, the above findings suggested the diagnosis of HPS. Whole exome sequencing was recommended for this patient, and in following its results, identified a homozygous frameshift variant in the TALDO1 gene, c.793del, p.Gln265fs. This confirmed the diagnosis of TD complicated with HPS.

The patient is currently at home, being followed in outpatient and receiving multidisciplinary care, along with home-based oxygen supplementation upon need. Additionally, the patient’s case was referred to liver transplant specialists for further discussion of the possibility of liver transplantation with metabolic medicine/genetics teams in hopes of improving her outcome.

## Discussion

This is a case of an eight-year-old girl known to have hepatosplenomegaly since early infancy, which was lost for follow-up, and revealed several years later to be a TD that was complicated with HPS. The rarity of TD and the non-adherence of parents to the diagnostic investigations have led to a delay in the diagnosis and the occurrence of severe complications with advanced functional symptoms that could threaten the life of the patient.

TD appears to be relatively prevalent in populations with high levels of consanguinity, mainly middle eastern ethnicity. This is shown in the example of the first case series published by Dr. Wafaa Eyaid, where she identified 12 patients identified in six Saudi families during the period from 2002 to 2010 [2]. Two further cases from Saudi Arabia were described later, one by Mukhtar et al. [6] and one by Alqoaer et al. [10]. In addition, four cases were reported by Al-Shamsi et al. from the United Arab Emirates [11]. More recently, Williams et al. performed a retrospective study on 34 patients from 25 families in Amsterdam, 32 of which were homozygous for a mutation in TALDO1 (Genbank # NM_006755; gi5803186), which concords with the high frequency of consanguinity [1]. Furthermore, a novel mutation causing a late-onset TD was identified in a patient born of consanguineous Turkish parents [12]. Taking into consideration the rarity of TD, the above data suggest a great concentration of the reported cases in the Middle East region, which may indicate an ethnic susceptibility besides consanguinity.

Regarding HPS, three previously published cases of HPS secondary to TD have been identified in the literature. The first case was reported in 2014 about a pediatric patient who had TD and was diagnosed with HPS by contrast-enhanced echocardiography [13]. The second case was presented in 2020, describing a girl from Saudi Arabia who had TD diagnosed by whole-exome sequence and who became oxygen dependent. In addition to chronic liver disease, she was diagnosed with HPS at the age of two years using a 99Technetium macro aggregated albumin perfusion scanning (99mTc-MAA scan) study [6]. Liver transplantation was not performed in the two previous cases. The third case was of a Turkish baby who was born with neonatal jaundice and pancytopenia without dysmorphic features [14]. The baby had radiological features of hepatosplenomegaly and intrapulmonary shunt. At the age of 11 months, the patient received a cadaver liver graft, but unfortunately developed ventricular fibrillation on the second postoperative day and did not survive.

To date, there is no cure for TD, and the management consists of non-specific treatment of associated defects, such as surgical correction of cardiac abnormalities, supportive symptomatic treatment including supplementation with calcium and vitamin D, red blood cells and platelets transfusions, albumin therapy, and variceal band ligation in case of esophageal varices [1,5]. The prognosis, therefore, depends on the management of disease complications mainly liver failure. Higher mortality rates are observed in neonatal-onset cases, reaching 27% within the age of six months when the disease is presented in the first week of life [1]. The only potentially lifesaving approach for HPS is liver transplantation as even in severe cases it was shown to prolong survival [9]. Stefanowicz et al. reported the case of two Polish brothers who had TD with liver nodular fibrosis indicating severe liver injury, in association with renal tubulopathy. Both brothers benefitted from liver transplantation, at the age of 11 and 14 years, with satisfactory functional outcomes for up to 23 months of post-transplantation follow-up [5]. Currently, the literature includes at least six cases of TD that have been transplanted (Table 2). However, liver transplantation does not reverse pulmonary vessel malformations [9] and may be of limited interest in the case of multi-organ dysfunction besides the risk of recurrence [5,10]. Moreover, TD-associated end-stage diseases expose to a high risk of peri- and postoperative complications following liver transplantation including graft failure, infection, and progressive renal failure, which imposes early and multi-disciplinary discussion to consider such procedure [5].

**Table 2:** Cases of liver transplantation in transaldolase deficiency LT: liver transplantation; GGTP: gamma-glutamyltranspeptidase; HPS: hepatopulmonary syndrome § liver transplantation was performed before the diagnosis of transaldolase deficiency.

Country (ref)	Gender, age at diagnosis	First presentation	Mutation	HPS	Age at LT	Outcome
Turkey [14]	Male, 5 months	Conjugated jaundice on the second day of life	Homozygote c.412C>T, p.(Arg138Ter)	+	11 months	Day 1 post-LT, the patient developed ventricular fibrillation and suddenly died of cardiac arrest.
Pakistan [14]	Female, 3 months	Hyperbilirubinemia (Conjugated), failure to thrive, coagulopathy, hepatosplenomegaly, and abdominal distension.	Homozygote c.695_696del p.(Ile232fs)	-	5 months	Her most recent follow-up in 2022, 21 years after LT, she has normal liver graft function and a normal echocardiogram.
Poland [5]	Male, 3 years	Bleeding diathesis with coagulopathy, deficiency of factors XI and XII, thrombocytopenia, and anemia.	Homozygote c.575G>A	-	14 years	At the age of 16 years, he had normal liver function tests including transaminase activity, GGTP, and bilirubin concentration, and no coagulopathy.
Poland [5]	Male, 5 months	Bleeding diathesis with coagulopathy, deficiency of factors XI and XII, thrombocytopenia and anemia, plus elevated transaminase activity.	Homozygote c.575G>A	-	11 years	Good general condition, liver function is preserved with normal transaminase, GGTP activity, normal bilirubin and albumin concentration, and no coagulopathy.
Emirates [11]	Female, 3 years	Hepatosplenomegaly, dysmorphic features, and cutis laxa at three months of age. Early-onset liver failure requiring transplantation.^§^	Homozygote c.574C>T, p.(Arg192Cys)	-	17 months	At the age of 3 years, she had a normal coagulation profile and slightly elevated liver enzymes. At the age of 10 years, she has normal liver graft function, moderate failure to thrive, and systemic hypertension.
Gambia [15]	7 months	Hepatocellular carcinoma within a background of cirrhosis with hepatitis, requiring transplantation.^§^	Homozygote c.512C>T (p.Ser171Phe)	-	16 months	Not documented

Like any inherited disorder, early diagnosis and familial screening for TD are paramount to provide a greater chance of disease slowdown and adequate familial planning. Williams et al. revealed the possibility of prenatal manifestations in TD, which may allow prenatal diagnosis among affected families [1]. Genetic counseling in the situation of consanguineous marriage is also necessary due to the autosomal recessive transmission of the disease. Our patient is a typical case of inadequate adherence to follow-up, leading to late diagnosis with advanced complications.

## Conclusions

Along with hepatic dysfunction, TD can cause serious multi-organ complications at a young age of life such as HPS. With little being known about this particular disease, our case adds new data and stresses the necessity of strict clinical, radiological, and functional follow-up of the respiratory status since the first establishment of genetic diagnosis regardless of the severity of the liver disease. Early liver transplantation combined with multi-disciplinary care would be an effective strategy to reduce the burden of the disease and improve the quality of life of the patients. Further research is warranted to better understand the pathological complexity of TD, and an open-source international registry may be a useful tool to advance the knowledge.

## References

[1] Williams M, Valayannopoulos V, Altassan R (2019). Clinical, biochemical, and molecular overview of transaldolase deficiency and evaluation of the endocrine function: update of 34 patients. J Inherit Metab Dis.

[2] Eyaid W, Al Harbi T, Anazi S (2013). Transaldolase deficiency: report of 12 new cases and further delineation of the phenotype. J Inherit Metab Dis.

[3] Verhoeven NM, Huck JH, Roos B (2001). Transaldolase deficiency: liver cirrhosis associated with a new inborn error in the pentose phosphate pathway. Am J Hum Genet.

[4] Xue J, Han J, Zhao X, Zhen L, Mei S, Hu Z, Li X (2021). Prenatal diagnosis of fetus with transaldolase deficiency identifies compound heterozygous variants: a case report. Front Genet.

[5] Stefanowicz M, Janowska M, Pawłowska J (2021). Successful liver transplantation in two Polish brothers with transaldolase deficiency. Children (Basel).

[6] Mukhtar G, Al-mobaireek K, Eltahir S, Alzaid MA, Alotaibi W (2020). Hepatopulmonary syndrome in a child with transaldolase deficiency: a case report. ECPRM.

[7] Shayota BJ, Donti TR, Xiao J (2020). Untargeted metabolomics as an unbiased approach to the diagnosis of inborn errors of metabolism of the non-oxidative branch of the pentose phosphate pathway. Mol Genet Metab.

[8] Lee-Barber J, English TE, Britton JF, Sobreira N, Goldstein J, Valle D, Bjornsson HT (2019). Apparent acetaminophen toxicity in a patient with transaldolase deficiency. JIMD Rep.

[9] Kim KY, Kim TH, Lee JM, Yi NJ, Kim HY, Moon JS, Ko JS (2021). Clinical outcomes and risk factors of hepatopulmonary syndrome in children. Sci Rep.

[10] Alqoaer K, Asaad Z, Halabi M (2019). Severe infantile transaldolase deficiency: a case report. J Adv Pediatr Child Health.

[11] Al-Shamsi AM, Ben-Salem S, Hertecant J, Al-Jasmi F (2015). Transaldolase deficiency caused by the homozygous p.R192C mutation of the TALDO1 gene in four Emirati patients with considerable phenotypic variability. Eur J Pediatr.

[12] Lafcı NG, Colak FK, Sahin G, Sakar M, Çetinkaya S, Savas-Erdeve S (2021). Hypergonadotrophic hypogonadism in a patient with transaldolase deficiency: novel mutation in the pentose phosphate pathway. Hormones (Athens).

[13] Jassim N, Alghaihab M, Saleh SA, Alfadhel M, Wamelink MMC, Eyaid W (2014). Pulmonary manifestations in a patient with transaldolase deficiency. JIMD Rep.

[14] Grammatikopoulos T, Hadzic N, Foskett P (2022). Liver disease and risk of hepatocellular carcinoma in children with mutations in TALDO1. Hepatol Commun.

[15] Leduc CA, Crouch EE, Wilson A (2014). Novel association of early onset hepatocellular carcinoma with transaldolase deficiency. JIMD Rep.

